# Antimicrobial effect of copper alloys on *Acinetobacter* species isolated from infections and hospital environment

**DOI:** 10.1186/s13756-018-0300-x

**Published:** 2018-01-22

**Authors:** Anna Różańska, Agnieszka Chmielarczyk, Dorota Romaniszyn, Grzegorz Majka, Małgorzata Bulanda

**Affiliations:** 1 Chair of Microbiology, Faculty of Medicine, Jagiellonian University Medical College, ul. Czysta 18, 31-121 Kraków, Poland; 2 Chair of Immunology, Faculty of Medicine, Jagiellonian University Medical College, ul. Czysta 18, 31-121 Kraków, Poland

**Keywords:** *Acinetobacter*, Hospital environment, Antimicrobial copper

## Abstract

**Background:**

An increased proportion of Gram-negative bacteria have recently been reported among etiologic agents of infection. In Poland, *Acinetobacter baumannii* is a big problem for hospitals, especially intensive care units. Touch surfaces made from materials with antimicrobial properties, especially copper alloys, are recommended as a supplementary method of increasing biological safety in the hospital environment.

**Aim of the study:**

The objective of this study is to determine the susceptibility to selected copper alloys of three clinical *Acinetobacter baumannii* strains, one *Acinetobacter lwoffi* and an *A. pittii* strain isolated from the hospital environment.

**Material and method:**

The modification of the Japanese Standard, which the ISO 22196:2011 norm was used for testing antimicrobial properties of CuZn37, CuSn6 and CuNi18Zn20 and Cu-ETP and stainless steel as positive and negative control, respectively.

**Results:**

The highest cidal efficiency, expressed as both time and the degree of reduction of the initial suspension density, against all of the tested *Acinetobacter* strains was found for ETP copper. But, the results of our study also confirmed effective activity (bacteriocidal or bacteriostatic) of copper alloys selected for the study, contrary to the stainless steel. The reduction in bacterial suspension density is significantly different depending on the strain and copper alloy composition.

**Conslusions:**

The results of our study confirmed the effective antibacterial activity of copper and its selected alloys against clinical *Acinetobacter baumannii* and *Acinetobacter lwoffii* strains, and *Acinetobacter pittii* strain isolated from the hospital environment.

## Introduction

An increased proportion of Gram-negative bacteria have recently been reported among etiologic agents of infection according to ECDC reports obtained under the international project [1]. Additionally, there is also a growing number of multidrug-resistant strains recorded in this group. In Poland, *Acinetobacter baumannii* is a big problem for hospitals, especially intensive care units [2, 3]. A significant issue associated with MDRAB (multidrug-resistant AB) is its great ability to survive in the abiotic environment [4]. The effectiveness of traditional disinfection methods, including the hand hygiene procedure conforming with WHO recommendations, is not infallible owing to human error or skipping the procedures in some situations. In literature, also some reports on reduced susceptibility to disinfectants and antiseptics in healthcare settings can be found [5]. Therefore, attempts are made to introduce various kinds of equipment made of materials with antimicrobial properties to hospitals, including those based on Cu^+^. Antimicrobial properties of numerous copper alloys have been certified by the Environmental Protection Agency (EPA). However, the EPA’s antimicrobial properties assessment procedure takes into account such microorganisms as methicillin-sensitive and methicillin-resistant *S. aureus*, *Enterobacter aerogenes*, *Pseudomonas aeruginosa* and *E. coli O157:H7* [6]. Only *S. aureus* and *E. coli* are designated in the recommendations contained in the which the ISO 22196:2011 norm for testing of antimicrobial properties of non-porous materials [7].

The objective of this study is to determine the susceptibility to selected copper alloys of three *Acinetobacter baumannii* strains (differing in terms of drug resistance including two clinical strains, isolated from invasive infection), one clinical *A. lwoffii* strain and an *A. pittii* strain isolated from the hospital environment.

## Material and methods

### Chosen copper alloys and their preparation

Metal samples measuring 2.5 cm × 2.5 cm with a thickness of 1–2.5 mm were provided by the Faculty of Non-ferrous Metals, AGH University of Science and Technology, Kraków. Before their delivery for microbiological testing, the samples underwent mechanical polishing, cleaning and degreasing by immersion in acetone. Prior to use for microbiological testing, the samples were sterilized by wiping with 96% alcohol. Studies were conducted on the following copper alloys: brass CuZn37, tin bronze CuSn6, nickel silver CuNi18Zn20, and for ETP copper (99.9% Cu) as a positive control (presumed highest antimicrobial efficacy) and stainless steel as a negative control (assumed lack of antimicrobial properties). The copper alloys selected for this study are the most well-known and most frequently used in various industries. The alloys used in the study with data on the concentration percentage of copper are listed in Table 1.

**Table 1:** Compositions (%) of the tested commercial copper alloys

Common name	UNS^*^ code	Cu	As	Bi	Cd	Fe	Mn	Al	Ni	P	Pb	Sb	Si	Sn	Zn
Copper Cu-ETP	C11000	99.9	0.0	0.001	0.001	0.002	0.001	0.0	0.0	0.030	0.002	0.000	0.008	0.0	0.0
Yellow Brass CuZn37	C27400	63.2	0.001	0.001	0.001	0.001	0.001	0.001	0.06	0.001	0.004	0.001	0.008	0.0	36.7
Phosphor Bronze CuSn6	C51900	94.1	0.006	0.002	0.0	0.001	0.001	0.016	0.01	0.222	0.038	0.001	0.002	5.5.	0.1
Nickel silver CuNi18Zn20	C75200	63.1	0.001	0.001	0.001	0.027	0.12	0.001	17.9.	0.001	0.001	0.008	0.001	0.001	18.9.
Stainless Steel	S30400	Fe 68.8, C 0.07, Cr 19, Mn 2, N 0.1, Ni 10, P 0.045, S 0.015, Si 1													

*UNS* (Unified Numbering System), *ETP* Electrolytic Tough Pitch

### Acinetobacter strains selected and their characteristics

The studies were carried out on five strains of the genus *Acinetobacter*, including three *A. baumannii* (AB) strains, one *Acinetobacter pittii* strain (AP, isolated from the hospital environment [8]) and one *Acinetobacter lwoffii* strain (ABLW). The strains employed differed in terms of their drug resistance, presence of genes detected and biofilm-forming potential.

Susceptibility of strains was tested using disk diffusion antimicrobial susceptibility methods on Mueller-Hinton agar plates according to the current guidelines of the European Committee on Antimicrobial Susceptibility Testing (EUCAST Tables v. 6.0; http://www.eucast.org v.6.0 accessed 1.12.2016). The following antimicrobials were tested (all discs were from Oxoid, Basingstoke, UK): ampicillin-sulbactam (SAM 20 μg), piperacillin-tazobactam (TZP 30 μg and 6 μg), cefepime (FEP 30 μg), ceftazidime (CAZ 10 μg), imipenem (IMP 10 μg), meropenem (MEM 10 μg), ciprofloxacin (CIP 5 μg), levofloxacin (LEV 5 μg), amikacin (AK 30 μg), gentamicin (CN 10 μg), tobramycin (TOB 10 μg), netilmicin (NET 10 μg), tetracycline (TET 30 μg), trimethoprim-sulfamethoxazole (SXT 1.25/23.75 μg).

The minimum inhibitory concentration (MIC) for colistin (range 0.016 to 256 μg/ml) and polymyxin B (range 0.016 to 256 μg/ml) was determined by the E-test (bioMerieux, Marcy l’Etoile, France). Colistin and polymyxinB MICs of ≤ 2 and ≥ 4 mg/L, respectively, were interpreted as susceptible and resistant according to the Clinical and Laboratory Standard Institute (CLSI) guidelines.

MDR strains were defined as those strains that were non-susceptible to one antimicrobial in at least three different antimicrobial classes. XDR strains were defined as those strains that were susceptible to no more than two antimicrobial classes [9].

Multiplex real-time PCR was used to screen for the four blaOXA genes and the blaVIM gene as described previously [2].

*A. baumannii* strain no. 835 was isolated from cerebrospinal fluid. This is an extensively drug resistant (XDR) strain. It was resistant to: penicillins with inhibitors (piperacillin/tazobactam, ampicillin/sulbactam), cephalosporins (ceftazidime and cefepime), carbapenems (imipenem and meropenem), fluoroquinolones (ciprofloxacin and levofloxacin), aminoglycosides (amikacin, tobramycin, gentamicin and netilmicin) and sulfamethoxazole with trimethoprim and tetracycline. In addition, the minimum inhibitory concentration for colistin (MIC = 1) and polymyxin (MIC = 0.19) was determined by E-test. Strain no. 835 belongs to the international clone II and has the blaOXA-23 and blaOXA24 genes [2].

*A. baumannii* strain no. 366 was also isolated from cerebrospinal fluid. This is an extensively drug resistant (XDR) strain. It was resistant to: penicillins with inhibitors (piperacillin/tazobactam, ampicillin/sulbactam), cephalosporins (ceftazidime and cefepime), carbapenems (imipenem and meropenem), fluoroquinolones (ciprofloxacin and levofloxacin), aminoglycosides (amikacin, tobramycin, gentamicin and netilmicin) and sulfamethoxazole with trimethoprim and tetracycline. MIC for colistin (MIC = 1.5) and polymyxin (MIC = 0.25) was determined by E-test. Strain no. 366 belongs to the international clone II and has the blaOXA24 gene [2].

*A. lwoffii* strain no. 91 was isolated from blood. This is an extensively drug resistant (XDR) strain. It was resistant to: penicillins with inhibitors (piperacillin/tazobactam), cephalosporins (ceftazidime), carbapenems (imipenem and meropenem), fluoroquinolones (ciprofloxacin and levofloxacin), aminoglycosides (amikacin, tobramycin, gentamicin and netilmicin) and sulfamethoxazole with trimethoprim. In addition, the minimum inhibitory concentration for colistin (MIC = 0.25) and polymyxin (MIC = 0.094) was determined by E-test. Strain no. 91 has the blaOXA24 and VIM genes [2].

*A. pittii* strain no. 70: (AB70) was isolated from the hospital environment [8]. It was resistant only to: ceftazidime, sulfamethoxazole with trimethoprim and tetracycline. MIC for colistin (MIC = 0.75) and polymyxin (MIC = 0.75) was determined by E-test.

*A. baumannii* strain no. BAA-1605 comes from ATCC collection and was isolated from sputum. It was resistant to penicillins (piperacillin/tazobactam, ampicillin/sulbactam), cephalosporins (ceftazidime and cefepime), carbapenems (imipenem and meropenem), fluoroquinolones (ciprofloxacin and levofloxacin), aminoglycosides (gentamicin). It was sensitive to amikacin and tobramycin. MIC for colistin (MIC = 0.75) and polymyxin (MIC = 0.38) was determined by E-test.

### Quantitative culture method to determine the antimicrobial effectiveness of copper and its alloys

We used a modification of the Japanese Standard, which the ISO 22196:2011 [7] norm is based on, recommended in Europe for testing of antimicrobial properties of non-porous materials. The bacterial suspension used to apply the metal alloys tested was prepared in TSB.

The tested bacterial strains were stored in glycerol at − 70 °C. One day before antimicrobial efficacy testing, a small amount of the suspension was taken form a frozen sample, inoculated onto solid Muller-Hinton agar (MHA, BIOCORP, Warsaw, Poland) (clean culture) and then incubated for 24 h at 37 °C. From the obtained culture, a suspension was prepared in saline at a density of 0.5 McFarland standard (controlled using a densitometer bioSan, Riga, Latvia). Subsequently, 100 μL of the suspension with a density of 0.5 McFarland standard was transferred to 900 μL of TSB. Each time, a control of the viability of the bacteria obtained in the culture on solid medium and the control of the precise initial concentration (its density expressed in CFU/mL) was performed.

Samples of the metals tested were placed in a sterile container made of PVC with a capacity of 100 mL that was 6 cm in diameter, and then, 100 μL of the test suspension was applied (the composition depended on the variant of the experiment). Next, the container was covered with sterile polypropylene foil measuring 2 cm × 2 cm to provide close contact between the bacterial suspension and the metal surface. The container was covered to prevent contamination of the sample with microbes from the air, but it remained loose enough that aerobic conditions were maintained throughout the course of exposure and when left for a specified period of time (0, 60, 120, 180, 240, and 300 min) at approx. 22 °C (room temperature).

After a certain period of time, 5 mL of the TSB solution and approx. 30 sterile glass beads that were 2 mm in diameter were placed into the container and shaken for 2 min in a shaker (shaker-incubator ES-20/60, Riga, Latvia). Then, 100 μL of the wash was collected, 4 serial decimal dilutions were prepared, of which 100 μL was inoculated onto solid MHA for each time-point. After a 24-h incubation, individual colonies were counted on the plates when the resulting number was countable.

For each metallic material, each exposure time for all microbes was repeated three times. To count the amount of CFU/mL after exposure of the bacterial suspension to the studied materials, the average of the triplicates was used. The formula for the calculation was *CFU*/*mL* = (*n* × *f* × *v*1)/(*v*2 × *v*3), where: *n* – average number of colonies/plate in dilution, f – dilution factor, v1 – volume of TSB used for washing the bacteria that survived after exposure, v2 – volume used and applied on metallic coupons, and v3 – volume of the plated material (v1–3 in mL).

To evaluate the effectiveness of the antimicrobial activity, the criteria used by Souli et al. [10] were adopted according to which a suspension density reduction occurred, ranging from ≤ 2 to < 3 log mean bacteriostatic properties, as well as a reduction of over 3 log – bactericidal properties.

Results of susceptibility tests for the copper alloys tested were shown as charts of CFU/ml values in chosen time periods and were expressed as the mean ± SEM. Two-way Anova with repeated measures analysis of variance was used to evaluate the effects of both time and strain for every tested alloy. In the second approach, for each tested alloy XDR strains were compared with non-XDR strains in a similar two-way Anova analysis (effects evaluated were time and XDR characteristic). *P* values less than 0.05 were considered statistically significant.

## Results

**Fig. 1:**
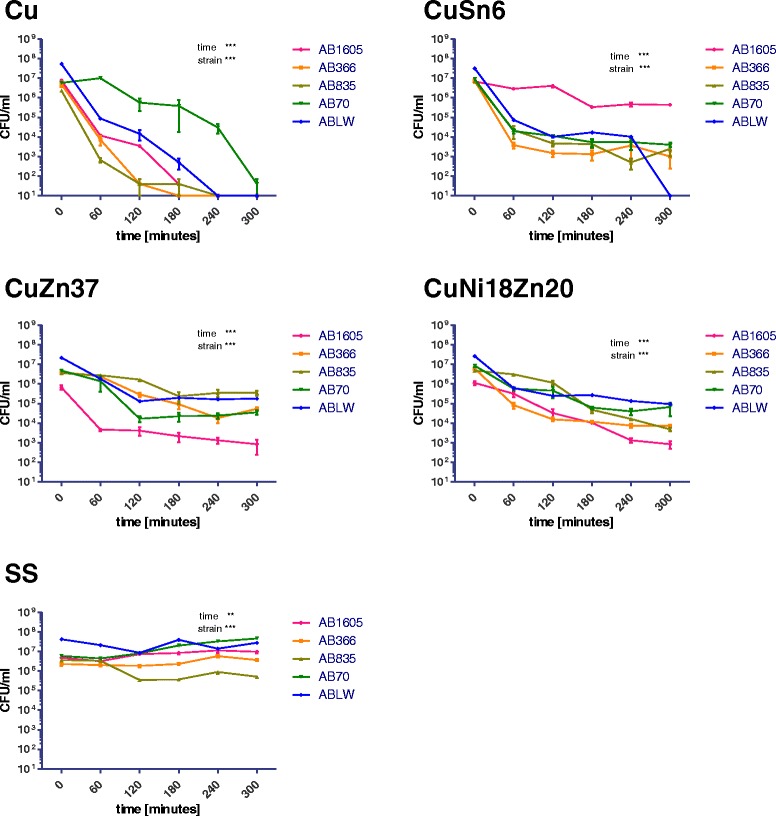
Tested bacteria inoculum density (CFU/mL) reduction on metallic materials in chosen time periods, average from of three repetitions for each time point. *** - *p* < 0.001, ** - *p* < 0.01, ns – not statistically significant

The highest cidal efficiency, expressed as both time and the degree of reduction of the initial suspension density, against all of the tested *Acinetobacter* strains was found for ETP copper. In this case, complete reduction from the level of around 10^7^ CFU/ml to zero was observed for AB strains and for *Acinetobacter lwoffii*. As regards the environmental AP strain, after 300 min there was a decimal log reduction of over 3 for the initial density, which confirms the bactericidal properties of copper against this strain. As for tin bronze, cidal activity was found after 180 min against two AB strains and the environmental AP strain (decimal log reduction of over 3) and bacteriostatic activity was shown after 60 min. For ABLW cidal activity of tin bronze was observed after 60 min. It was only for the ATCC1605 strain that the observed level of reduction within 300 min did not exceed 2 log. For brass, bacteriostatic properties were confirmed for two strains: one clinical AB strain and the environmental AP strain. As for ATCC1605, bacteriostatic properties of brass were demonstrated, while for the drug-resistant AB and ABLW strains, reduction of the initial suspension density did not exceed 2 log. As regards new silver, for two strains, i.e., AB ATCC1605 and one clinical AB strain, bactericidal properties were confirmed, and for one of clinical AB and environmental AP strains bacteriostatic activity was demonstrated. With respect to stainless steel, no *Acinetobacter* strain exhibit reduction of the initial suspension density after 300 min that would meet the bacteriostatic criteria, i.e., that of over 2 log. The results obtained are presented in Table 2 and Fig. 1.

**Table 2:** Tested bacteria inoculum density (CFU/mL) reduction on Cu-ETP, CuSn6, CuZn37, CuNi18Zn20, stainless steel (S. steel), in chosen time periods (T, in minutes)

CuZn37					
Time	AB1605	AB366	AB835	AB70	ABLW
T0	6.67E + 05	4.17E + 06	3.58E + 06	4.92E + 06	2.22E + 07
T60	4.67E + 03	2.47E + 06	2.78E + 06	1.40E + 06	1.80E + 06
T120	4.17E + 03	2.90E + 05	1.67E + 06	1.67E + 04	1.32E + 05
T180	2.17E + 03	9.17E + 04	2.33E + 05	2.27E + 04	1.95E + 05
T240	1.33E + 03	1.83E + 04	3.50E + 05	2.42E + 04	1.63E + 05
T300	8.33E + 02	5.33E + 04	3.50E + 06	3.57E + 04	1.80E + 05
CuSn6					
T0	6.83E + 08	7.50E + 06	7.17E + 06	9.00E + 06	2.93E + 08
T60	2.92E + 06	3.83E + 03	2.23E + 04	2.00E + 04	7.50E + 04
T120	3.33E + 05	1.50E + 03	4.67E + 03	1.13E + 04	1.43E + 04
T180	4.67E + 05	1.33E + 03	4.33E + 03	5.33E + 03	1.73E + 04
T240	4.33E + 05	3.67E + 03	5.00E + 02	5.33E + 03	1.02E + 04
T300	4,33E + 05	1.00E + 03	2.50E + 03	4.00E + 03	0.00E + 00
CuNi18Zn20					
T0	1.13E + 06	6.05E + 06	4.90E + 06	8.67E + 06	2.68E+07
T60	3.17E + 05	8.33E + 04	3.13E + 06	5.80E + 05	2.48E+06
T120	3.28E + 04	1.58E + 04	1.15E + 06	4.50E + 05	2.43E+05
T180	1.02E + 04	1.20E + 04	4.80E + 04	6.17E + 04	2.72E+05
T240	1.33E + 03	7.50E + 03	1.62E + 04	4.00E + 04	1.37E+05
T300	8.33E + 02	7.33E + 03	4.83E + 03	6.67E + 04	1.22E+05
Cu					
T0	7.67E + 06	5.17E + 06	2.37E + 06	5.80E + 06	5.45E+07
T60	1.17E + 04	7.33E+03	6.67E+02	1.01E + 07	8.83E+04
T120	3.50E + 03	6.67E + 02	3.33E + 02	5.62E+05	1.67E+04
T180	3.33E + 02	0.00E + 00	1.50E + 03	3.85E + 05	5.00E+02
T240	1.00E + 00	0.00E + 00	0.00E + 00	3.00E + 04	0.00E+00
T300	1.00E + 00	0.00E + 00	0.00E + 00	1.00E + 03	0.00E+00
Stainless steel					
T0	4.83E + 06	2.33E + 06	3.65E + 06	6.00E + 06	4.27E + 07
T60	3.17E + 06	2.00E + 06	3.32E + 06	4.50E + 06	2.15E + 07
T120	7.33E + 06	1.83E + 06	3.52E + 05	8.00E + 06	8.00E + 05
T180	8.50E + 06	2.33E + 06	3.73E + 05	2.00E + 07	1.30E + 07
T240	1.13E + 07	5.83E + 06	8.98E + 05	3.30E + 07	1.45E + 07
T300	9.83E + 06	3.67E + 06	5.10E + 05	4.62E + 07	2.82E + 07

An interesting observation is that the XDR strains seem more susceptible to the antibacterial effect of copper and tin bronze. These differences, however, are not statistically significant. However, the trend is reversed for two other tested copper alloys – for both brass and nickel silver, the XDR strains exhibit higher resistance to cidal effects of the alloys in comparison to non-XDR strains. What is more, in case of brass the observed differences between XDR and non-XDR strains were statistically significant.

## Discussion

Among the tested materials made of copper and its alloys, the most effective antibacterial activity was found for copper, followed by tin bronze, while the weakest was shown for brass and nickel silver. Despite finding statistically significant differences for the bacterial suspension density at the tested time points for individual strains, no bactericidal or bacteriostatic properties were observed for stainless steel. These findings are consistent with the results obtained using the same methodology for *S. aureus*, *E. coli*, and coagulase-negative staphylococci [11, 12].

Greater discrepancies were observed for individual strains exposed on particular metal materials, however, no evident relationship was noted for strain/copper alloy variables. For instance, only for the AP strain on copper, complete reduction was not observed after 300 min, although an approx. 5 log reduction was demonstrated. For this strain, the decrease in suspension density after 240 min did not exceed 3 log, while for the remaining ones, the decrease in suspension density of about 3 log was observed as soon as after 60 to 180 min. The AP strain was isolated from the hospital environment, in contrast to the others, which were isolated from materials coming from patients. But the results for other materials and this strain were different – the rate and degree of reduction for this strain were comparable to the other strains tested. As regards tin bronze and nickel silver, the greatest antibacterial activity (border of bactericidal properties, approx. 3 log reduction) was observed for the AB model strain, while in the case of tin bronze, the reduction in density of the initial bacterial suspension did not exceed 2 log. So, apart from the generally observed regularities, exceptional cases were observed, which indicates that research in this field should be continued. Our studies were carried out using a suspension containing a single species, and separate testing for selected species is part of the existing norms and guidelines [6, 7]. In real life, in the case of contamination with material containing a mixture of different bacterial strains/species, the results can be difficult to predict, which also requires further laboratory and clinical tests. On the other hand, most of isolates obtained from touch surfaces in Polish hospital wards were single strain isolates [8].

Generally, the antimicrobial effectiveness of copper alloys depends proportionally on the copper content in a given alloy [11]. Antimicrobial properties of these materials are also variable depending on the type of exposure (wet or dry), the presence of organic contamination or lack thereof, ambient temperature or humidity [13]. These studies were carried out under identical conditions as for the previously tested methicillin-resistant *Staphylococcus aureus* strains, *Escherichia coli* and four coagulase-negative staphylococci strains with different profiles of drug resistance and biofilm-forming potential [11, 12]. All of the strains tested, belonging to different bacterial species, were found to possess statistically significant differences as regards the speed and degree of reduction of initial bacterial suspension density both for various bacterial strains, as well as copper alloys. The results of studies performed beforehand for *Staphylococcus aureus*, *Escherichia coli* and coagulase negative staphylococci (under the same conditions) [11, 12], and for *Acinetobacter* strains, showed the following regularities: the greatest antimicrobial effectiveness for Cu-ETP, and among the alloys tested here – the lowest for CuZn37. However, with isolated exceptions, all alloys demonstrated bacteriostatic or bactericidal properties within the time limit not exceeding 300 min. From the clinical point of view, implementing copper alloys with confirmed bacteriocidal or bacteriostatic properties against *Acinetobacter* as a material for touch surfaces in hospital wards should be very important, because there is a problem of infections and epidemics caused by bacteria of this genera in Polish hospitals [2, 3].

In the framework of this project we started investigating the antimicrobial properties of copper alloys for application as touch surfaces in healthcare facilities, in two experiment variants for selected model *E. coli* and *S. aureus* strains [11]. One made use of bacterial suspensions in saline, as a simulation of the environment without organic contamination, the other one used TSB broth as a simulation of the environment with organic pollutants. In the first variant, a complete reduction was obtained in over 10 min compared to the bacterial suspension in TSB, demanding longer time for reduction. In the tests using *Acinetobacter* strains, only a suspension in TSB was employed, in order to confirm the degree of reduction precisely in the conditions simulating organic contaminations, more conducive for the survival of bacterial cells. It is worth mentioning that the study conditions were more restrictive for materials made of copper alloys, and more conducive to the survival or multiplication of bacteria, than the conditions recommended by EPA or the so-called Japanese Standard [6, 7].

The sensitivity of the strain from the genus *Acinetobacter* to copper and brass CuZn37 was also tested by Souli et al. [10]. The authors recorded similar results for brass as the ones obtained by us for one clinical AB and environmental AP strains (bacteriostatic activity within 300 min). Worse results were obtained for copper, as in the case of the strain tested by them, a reduction of around 3 log was observed within 300 min (reduction of around 5 log was observed after 24 h). The remaining strains tested by them and belonging to the Gram-negative bacterial species (*E. coli*, *Enterobacter spp*., *Klebsiella pneumoniae* and *Pseudomonas aeruginosa*), were more sensitive to both copper and brass.

Steindl et al. carried out a study which tested antimicrobial effect of copper on multidrug-resistant bacteria, including Gram-negative bacilli such New Delhi metallo-beta-lactamase-1 (NDM-1) producing *K. pneumoniae* and extended spectrum β-lactamase (ESBL)-type cefotaxime-resistant-Munich (CTX-M) producing *E. coli* [14]. They have found complete reduction of the initial bacterial suspension density from around 1.5 × 10^8^ after 60 min for NDM-1 *K. pneumoniae* and after 2 h for ESBL CTX-M *E. coli*. In our study/experiment, in the case of Cu-ETP, complete reduction of bacterial suspension density was obtained after 180 to 240 min for three out of four strains tested. Warnes et al. reported complete reduction of bacterial suspension of *E. coli O157:H7* and *Salmonella typhimurium* in time not exceeding 30 min [15]. But, in the cited experiment, Warnes et al. applied only 1 μl of suspension of the density of 10^7^ on plates measuring 1 cm and it was dry exposure. Therefore, the conditions were rather hardly comparable to the ones used in our study.

And the efficiency of the antimicrobial activity of copper strictly depends on the conditions of the experiment – the temperature, the method for preparing the suspension, and exposure conditions (dry vs. wet), which is confirmed by other authors [16–18]. However, when copper is used as a material for touch surfaces, the *contact killing* mechanism is pointed out as the key instrument for antimicrobial properties of these materials. Laboratory tests indicate that the starting point of *contact killing* is due to dissolved copper ions in the medium acting on the surface of copper causing cell alterations [19]. In the studies on the mechanism of the copper influence on *E. coli*, Hong et al. [20] demonstrated that *contact killing* is triggered by non-enzymatic oxidative damage of membrane phospholipids, resulting in the loss of membrane integrity and cell death. Generally, the antibacterial effect of copper is related to its ability to release copper ions. Many studies attribute the antibacterial activity of copper to the capacity of the released ions to cause a great oxidative stress by producing reactive oxygen species in aerobic conditions. As a result, the first stage is a cell membrane degradation which allows copper ions to penetrate into the cell and damage lipids, proteins, nucleic acids and eventually to destroy the whole genetic material [21]. Destruction of genetic material is especially important in the context of growing drug resistance of bacteria causing hospital-acquired infections.

The results obtained, in conjunction with the data available in the literature from other studies, allow to assume the fact that the introduction of touch surfaces into hospital units should result in their reduced contamination with pathogenic microorganisms, including bacteria from the genus *Acinetobacter.* Such surfaces may complement the traditional methods for disinfection. In some cases, the implementation of such surfaces may involve the necessity of choosing an optimum disinfectant for the surface for maximum antimicrobial efficacy [22]. Susceptibility of copper alloys to oxidation may be the reason behind the reservations about introducing these materials into patient rooms; however, oxidation only reduces the esthetic appeal, but does not affect the reduction of antimicrobial properties, which was confirmed by both other authors and within the framework of this project [23, 24], which should be a superior value in this case.

### Limitations of the study

The performed study has several limitations. The degree of reduction of the bacterial suspension density was examined for five time points between 60 and 300 min (the time points were chosen subjectively), hence, the results do not provide any information on the degree of reduction for shorter or longer time periods. On the other hand, conditions applied in the experiment – wet exposure, suspension in TSB, simulating organic impurities, and high initial density of the bacterial suspension – enabled us to assess the potential for antimicrobial properties in relation to the Acinetobacter strains studied and this precise result was achieved. Another limitation of this study is the small number of strains from the genus Acinetobacter and the application of only one variant of exposure (i.e., wet). Due to the clinical significance of the Acinetobacter strains, research using these species should be continued, taking into account other strains and parameters influencing the sensitivity/resistance to copper alloy materials.

## Conclusions

The results of our study confirmed the effective antibacterial activity of copper and its selected alloys against clinical *Acinetobacter baumannii* and *Acinetobacter lwoffii* strains, and *Acinetobacter pittii* strain isolated from the hospital environment.

The reduction in bacterial suspension density is significantly different depending on the strain and copper alloy composition.

## Abbreviations

AB: *Acinetobacter baumannii*
ABLW: *Acinetobacter lwoffi*
AP: *Acinetobacter pittii*
ATCC: American Type Culture Collection
CFU: Colony forming units
EPA: Environmental Protection Agency
ETP: Electrolytic Tough Pitch
EUCAST: The European Committee on Antimicrobial Susceptibility Testing
MIC: Minimal inhibitory concentration
XDR: Extensively drug resistance
